# Cavernous Sinus Syndrome in a Polio-Afflicted Patient With Multiple Aneurysms

**DOI:** 10.7759/cureus.60673

**Published:** 2024-05-20

**Authors:** Devaun M Reid, Nishanth Chalasani, Monica Khadka, Sunny Kahlon, Martin Giangreco

**Affiliations:** 1 Internal Medicine, University of South Florida Morsani College of Medicine, Tampa, USA

**Keywords:** post-polio paralysis, pericallosal aneurysm, internal carotid artery aneurysm, neuro-surgery, cavernous sinus aneurysms

## Abstract

Cavernous sinus syndrome (CSS) is a complex, multifactorial condition that presents with a myriad of signs and symptoms including ptosis, double vision, and headache. We present the case of a 65-year-old woman with a chief concern of left-eye pain, including polio syndrome and hip replacement surgery. Unlike typical CSS cases often linked to tumors, this patient's condition involved a carotid-cavernous fistula (CCF), multiple internal carotid artery aneurysms, and a pericallosal aneurysm, without any associated tumor. She presented with severe left eye pain, ptosis, double vision, vomiting, headache, and other neurological symptoms since she woke up. Her treatment at a tertiary care center included diagnostic imaging, a cerebral angiogram, and embolization procedures, and she was discharged in stable condition. This case adds significant value to the medical literature by documenting the successful management of CSS with multiple aneurysms and a CCF, highlighting the importance of personalized treatment strategies and the effectiveness of modern embolization techniques in complex neurological conditions.

## Introduction

Cavernous sinus syndrome (CSS) is a condition characterized by various symptoms such as ptosis, double vision, and headache. Diagnosing and treating CSS is challenging due to its diverse manifestations in patients, which are often linked to underlying issues like carotid-cavernous fistulas (CCFs) and aneurysms in the internal carotid artery (ICA). Tumors are typically the most common cause of CSS [1]. This report discusses a unique instance of left-sided CSS accompanied by a CCF, several ICAs aneurysms, and a pericallosal aneurysm. Despite the severity of the condition, the patient was ultimately discharged in a stable state. This case contributes to the existing body of knowledge by illustrating a rare presentation of CSS.

## Case presentation

We present the case of a 65-year-old Hispanic woman afflicted with polio and a partial hip replacement on the left side not related to diabetes. She arrived at the emergency room experiencing severe pain in her left eye, drooping eyelid (ptosis), double vision, vomiting, and a headache concentrated in the area behind her left eye and the left frontal region since she woke up. Additionally, she reported experiencing mucous discharge from her left nostril, an unusual sensation in her left hand, numbness in the left frontal area, and pain in the area above her left eye (supraorbital region) since the morning as well.

**Figure 1:**
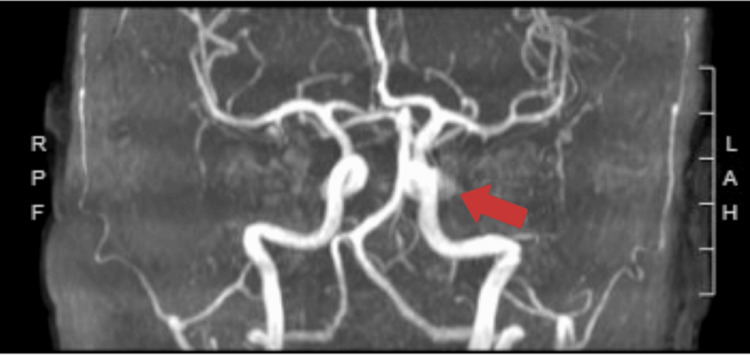
A 0.9 x 0.7 cm saccular aneurysm of the left cavernous segment of the left internal carotid artery.

**Figure 2:**
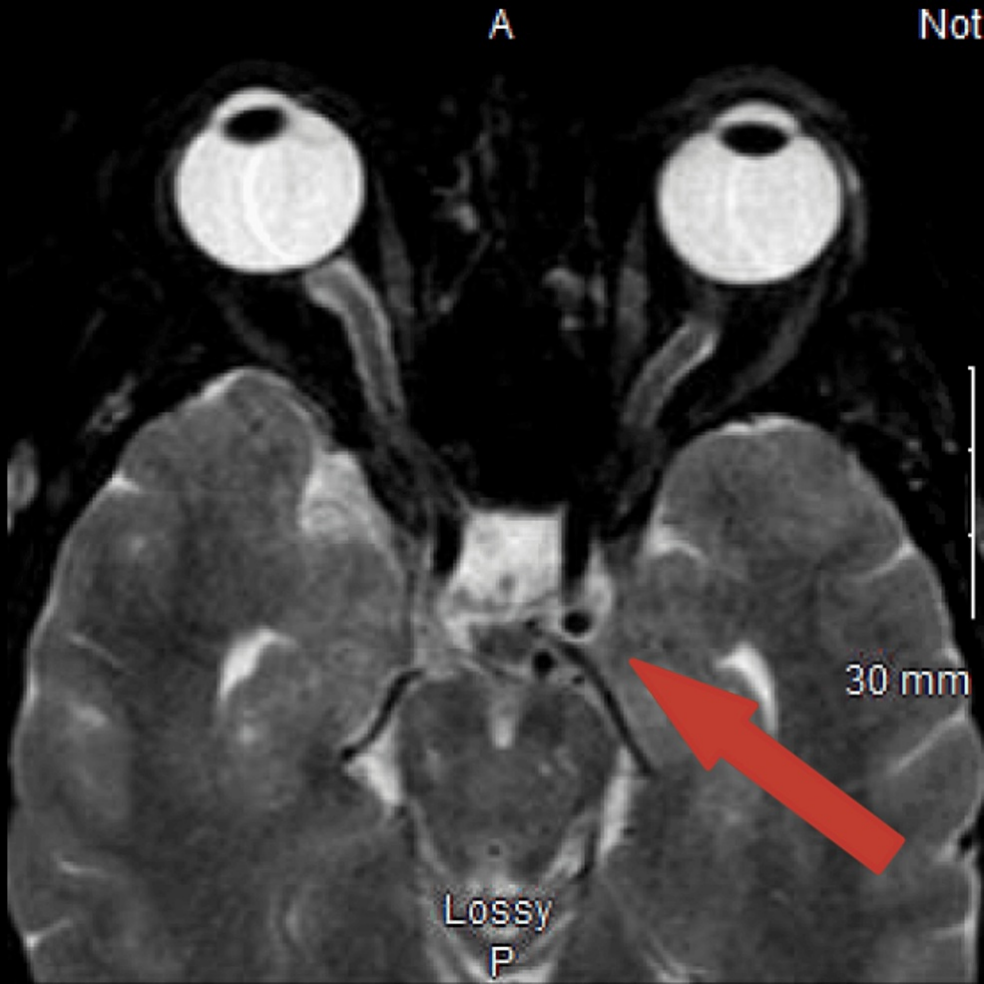
The left cavernous sinus is expanded and an abnormal flow void is visualized within the left cavernous sinus.

She was promptly admitted to the hospital on the same day, where initial examinations in the emergency department revealed a blood pressure of 135/64 mmHg with no audible bruits. She received immediate treatment with 4 mg of ondansetron, 2 mg of magnesium, 25 mg of intravenous (IV) diphenhydramine, and 650 mg of oral acetaminophen. Concerns about her condition led to a CT scan of the head without contrast, aimed at ruling out structural lesions such as subarachnoid hemorrhage and intracranial hemorrhage. Following this, an MR angiogram of the head and neck without contrast (Figure 1) and an MRI of the orbits without contrast (Figure 2) were conducted to further investigate her symptoms.

The findings were critical as the patient underwent a diagnostic cerebral angiogram (DCA) and digital subtraction angiography (DSA) embolization procedure to address a ruptured left ICA cavernous aneurysm with a surpass flow diverter and coils. Two days later, she required additional embolization of the fistula through the left superior ophthalmic artery. Her medical team meticulously managed her case, balancing the intricacies of her condition with the necessary interventions.

Throughout her hospitalization, she remained neurologically stable, a testament to the effective management and care she received. She was started on 2 mg oral dexamethasone for postprocedure care and prescribed 325 mg aspirin and 90 mg oral ticagrelor for ongoing management.

Her neurological examination, the day before discharge, revealed an average blood pressure of 91.3 mmHg. The neurological exam reveals a Glasgow Coma Scale score of 15, with the patient being alert and oriented. The patient is fluent in Spanish and communicates effectively through an interpreter. Neurological deficits include a left partial palsy of cranial nerves 3 and 4, and a complete palsy of cranial nerve 6, accompanied by left eye ptosis. Both pupils are reactive. The motor function is full in both upper extremities and the right lower extremity at 5/5, but diminished in the left lower extremity at 3/5. Sensory evaluation and coordination, including the finger-to-nose test, are intact. Her discharge the next day marked the end of an acute phase of medical intervention, culminating in her successful embolization for the left CSS, the rupture of her left ICA cavernous aneurysm, and the resulting CCF. She left the hospital in stable condition, with plans for a follow-up DCA scheduled in six weeks, a critical step in ensuring the continuity of her care and monitoring her recovery.

## Discussion

The presence of the left CSS alongside multiple aneurysms, notably a ruptured left ICA aneurysm leading to a CCF, presents a distinctive scenario. This complexity significantly enhances the case's value in medical literature, especially in the realms of diagnosis and management.

CCFs are abnormal connections between the carotid artery and the cavernous sinus, often caused by trauma or occurring spontaneously. They can lead to vision loss and are diagnosed primarily through cerebral angiography. Treatment usually involves endovascular embolization. Studies highlight the importance of monitoring for growth in related cavernous ICA aneurysms, especially larger ones [2-9].

The decision to manage the patient’s condition through embolization using flow diverters and coils was critical, considering her previous medical history. This approach aligns with the findings of a systematic review that highlighted the efficacy of flow diverters as a solitary treatment or adjunctive treatment in all patients with diagnosed CCFs [10]. Furthermore, the findings from this case reinforce a study that found that endovascular treatments, including the use of detachable balloons and coils, were effective and safe treatments for most spontaneous direct CCFs [11]. 

Moreover, the patient’s postprocedure period was notable for its stability, with no development of complications like deep vein thrombosis or sepsis, which are potential risks following such interventions. This is particularly remarkable considering the overall complication rate of 17.0% associated with the use of flow-diverting devices, with neurological morbidity being 4.5% [12]. The absence of these complications in a patient with a significant history of immobility due to polio contributes valuable information to postoperative care literature.

This case illustrates the importance of current advancements in neurosurgical treatments and decision-making. The successful use of both transarterial and transvenous embolization, as well as the pipeline embolization device (PED) for treating challenging intracranial aneurysms, highlights the advancements in treating complex neurovascular conditions [13]. Additionally, the neurosurgical team's cautious approach postprocedure, despite residual CCF, showcases the nuanced and complex decision-making in neurosurgery. This case serves as an important example of how cognitive biases can influence decisions and underscores the need for awareness and strategies to enhance decision-making in neurosurgery [14].

The significance of this case lies in its demonstration of how tailored endovascular strategies, as highlighted in the study on severe atherosclerotic stenosis of the intracranial ICA, can be effectively applied in complex neurosurgical scenarios [15]. The challenges posed by the location and tortuosity of the patient's aneurysms required a similar approach, emphasizing the need for individualized treatment modalities. Furthermore, the case provides valuable insights into managing neurosurgical patients with a history of polio and postpolio syndrome (PPS), given that there isn't a known direct link between polio and CCF [16]. The impact of PPS on the nervous system, leading to issues like muscle weakness and respiratory impairment, underscores the importance of adapting surgical and postoperative care strategies. Thus, this case serves as an important learning opportunity for understanding the intricacies of neurosurgical treatments for patients with unique medical histories.

## Conclusions

This case study highlights the intricate interplay of neurovascular pathology with a background of polio, offering significant insights into individualized neurosurgical treatments. The application of modern embolization techniques using flow diverters and coils effectively managed CCFs in a complex setting, demonstrating the benefits of customized intervention strategies. This approach not only aligns with existing literature on the efficacy of these techniques but also underscores the importance of tailored treatment in managing neurosurgical conditions.

Furthermore, the successful outcome of this case contributes to the broader understanding of CSS, especially in a patient with polio-induced immobility. The absence of typical postoperative complications, such as deep vein thrombosis or sepsis, provides valuable data on postoperative care and potential risks in neurosurgical procedures. The decision-making process reflected the challenges of neurosurgical practices where evidence-based guidelines are scarce, highlighting the need for meticulous clinical judgment and the importance of recognizing cognitive biases to enhance patient outcomes.
